# Haplotype Analysis Sheds Light on the Genetic Evolution of the Powdery Mildew Resistance Locus *Pm60* in *Triticum* Species

**DOI:** 10.3390/pathogens12020241

**Published:** 2023-02-02

**Authors:** Xuhui Huang, Xueli Jin, Xiaojie Ren, Wenxuan Wu, Wenjun Ji, Lihua Feng, Bo Jiang, Ming Hao, Shunzong Ning, Zhongwei Yuan, Lianquan Zhang, Bihua Wu, Dengcai Liu, Zhen-Zhen Wei, Lin Huang

**Affiliations:** 1 Triticeae Research Institute, Sichuan Agricultural University, Wenjiang 611130, China; 2 Institute of Plant Protection, Sichuan Academy of Agricultural Sciences, Chengdu 610061, China; 3 State Key Laboratory of Crop Gene Exploration and Utilization in Southwest China, Sichuan Agricultural University, Wenjiang 611130, China

**Keywords:** wheat powdery mildew, *Pm60*, allelic variants, domesticated wheat

## Abstract

Wheat powdery mildew (*Blumeria graminis* f. sp. *tritici*, *Bgt*, recently clarified as *B. graminis* s. str.), is one of the most destructive diseases of wheat. *Pm60* is a nucleotide-binding leucine-rich repeat (NLR) gene that confers race-specific resistance to *Bgt*. Allelic variants (*Pm60*, *Pm60a*, and *Pm60b*) were found in *Triticum urartu* and *T. dicoccoides*, the wild progenitors of wheat. In the present study, we studied the diversity of the *Pm60* locus in a large set of wheat germplasm and found 20 tetraploid wheats harboring the *Pm60* alleles, which correspond to three novel haplotypes (HapI–HapIII). HapI (*Pm60* allele) and HapII (*Pm60a* allele) were present in domesticated tetraploid wheats, whereas HapIII (*Pm60a* allele) was identified in wild tetraploid *T. araraticum*. A sequence comparison of HapII and HapIII revealed that they differed by three SNPs and a GCC deletion. Results of the phylogenetic analysis revealed that HapII was more closely related to the functional haplotype *MlIW172*. Infection tests showed that HapII-carrying lines display a partial resistance response to *Bgt#GH*, while HapI was susceptible. Our results provide insights into the genetic evolution of the *Pm60* locus and potential valuable alleles for powdery mildew resistance breeding.

## 1. Introduction

Wheat powdery mildew (*Blumeria graminis* f. sp. *tritici*, *Bgt*), recently clarified as *B. graminis* s. str. [1], is one of the most destructive foliar diseases that pose severe yield loss during the past decades worldwide [2]. Planting of *Bgt*-resistant cultivars is the most effective and environmentally safe strategy to control this disease [3]. However, wheat cultivars are prone to lose their resistance due to the rapid evolution of the pathogen and the emergence of new virulent *Bgt* isolates [4]. To date, over 69 loci for resistance to powdery mildew (*Pm1*–*Pm69*) have been formally documented and genetically mapped on different chromosomes in wheat [5,6]. Several *Pm* genes were identified from wheat wild relatives, such as *Triticum urartu* [7], *T. dicoccoides* [8], and *Aegilops biuncialis* [9].

Powdery mildew resistance gene *Pm60* was identified from *T. urartu* accession PI428309 and characterized as nucleotide-binding leucine-rich repeat (NLR) protein [7]. Three allelic variants were found in *T. urartu* populations and designated as *Pm60*, *Pm60a*, and *Pm60b*. The coding region of *Pm60a* has a 240-nucleotide deletion, whereas *Pm60b* contains a 240-nucleotide insertion, as compared with the *Pm60* sequence in PI428309, which resulted in the loss or addition of two of the leucine-rich repeat (LRR) motifs of *Pm60*. Although the deletion or insertion did not affect the resistance to *Bgt* isolate E09 [7], the deletion of the two LRR motifs in *Pm60a* substantially narrowed the resistance spectrum [10]. For example, *Pm60* was highly resistant to 43 out of 54 *Bgt* isolates tested, whereas *Pm60a* was only resistant to 5 of them [10]. A nonfunctional allele of *Pm60a*, designated as *Pm60a’*, was presented in multiple susceptible *T. urartu* accessions. The *Pm60a’* sequence was 98.52% identical to *Pm60a*, with 58 single nucleotide polymorphisms (SNPs) and a 3-nucleotide deletion [11].

The wild emmer wheat (*T. dicoccoides*) harbors the *Pm60* allele (*TdPm60*), which is an ortholog of *Pm60* from *T. urartu*. *TdPm60* was found to be present in the wild emmer donor lines of resistance genes *PmG16* [8], *MlWE18* [12], *MlIW72* [13], and *MlIW172* [14]. Among them, *MlWE18* and *MlIW172* were confirmed to be the functional alleles of *Pm60* by using ethyl methanesulfonate (EMS) mutagenesis and genetic transformation [12,14]. *TdPm60* constitutes a strong candidate for *PmG16* mildew resistance [8]. The results of the haplotype analysis of *Pm60* alleles revealed diversifications in sequence variation in the locus and presence/absence polymorphisms in wild emmer wheat populations [14]. *MlWE18* and *MlIW172*, together with additional eight haplotypes (Hap1–Hp8) of *TdPm60*, were defined in wild emmer [14], which shared four common single nucleotide variations between the *Pm60* alleles from *T. dicoccoides* and *T. urartu*. A different resistance spectrum of functional *Pm60* alleles was observed when inoculated with 20 *Bgt* isolates sampled from different locations of China [14], implying that these *Pm60* alleles and haplotypes are promising for breeding powdery mildew resistance wheat cultivars.

Previous studies focused on the characterization of *Pm60* alleles in either diploid *T. urartu* or tetraploid *T. dicoccoides* accessions. Whether there are further unidentified *Pm60* alleles in other wheat species has not been thoroughly assayed. In the present study, we screened a large collection of wheat germplasm with *Pm60* gene-based primers. This screening revealed that 20 genotypes harbor one of the *Pm60* alleles. The results of the sequence analysis identified three novel *Pm60* haplotypes from tetraploid wheats. Our results shed light on the evolution of the *Pm60* locus and provide potential valuable alleles for powdery mildew resistance breeding.

## 2. Materials and Methods

### 2.1. Plant Materials

Two hundred thirty-eight genotypes of different wheat species (Table S1) were used in the current study, including diploid *Ae. searsii*, *Ae. bicornis*, and *Ae. sharonensis*; tetraploid sources *T. timopheevii*, *T. armeniacum*, *T. carthlicum*, *T. dicoccon*, *T. dicoccoides*, *T. durum*, *T. palaeocolchicum*, *T. polonicum*, *T. turanicum*, and *T. turgidum*; as well as hexaploid wheat *T. spelta*, *T. sphaerococcum*, *T. aestivum*, *T. compactum*, *T. macha*, *T. vavilovii*, and *T. zhukovskyi*. The seeds of those wheat accessions were originally obtained from the National Small Grains Collection (NSGC, USDA) (PI and CItr numbers) and Triticeae Research Institute, Sichuan Agricultural University, Chengdu, China (AS numbers). 

### 2.2. Genomic DNA Extraction and Marker Analysis

Wheat seeds were wrapped in a moist filter paper and put in a chamber at a temperature of 4 °C for 3 days and transferred into a growth chamber at a temperature of 10 °C during the dark period (8 h) and 20 °C during the light period (16h) for 10 days. Total genomic DNA was prepared from seedling leaves using the cetyltrimethylammonium bromide (CTAB) method as described by Zhang et al. [15]. The DNA samples were checked for integrity by 0.8% agarose gel electrophoresis, followed by staining with GelRed Nucleic Acid Gel Stain (Biotium, Hayward, CA, USA). DNA concentration was assessed using a NanoDrop One spectrophotometer (Thermo Fisher Scientific, Waltham, MA, USA). Each PCR mixture (20 μL) contained 80–100 ng of template DNA, 300 nM of each of forward and reverse primers, and 10 μL of Taq Master Mix (P112-02, Vazyme Biotech Co., Nanjing, China). PCR amplification with the functional molecular marker for *Pm60* (*M-Pm60-S1*) [11] was programmed as 94 °C for 5 min, 35 cycles of 94 °C for 35 s, 57 °C for 35 s, and 72 °C for 1 min, terminated after an extension at 72 °C for 7 min. Alternatively, the high-fidelity ExTaq polymerase (Takara, Dalian, China) was used for the amplification of the entire *Pm60* region. Touchdown PCR amplification was carried out as described by Huang et al. [16]. PCR products were separated using 1.2% agarose gel electrophoresis, stained with GelRed Nucleic Acid Gel Stain (Biotium).

### 2.3. Sequence Analysis of Pm60

**Figure 1:**
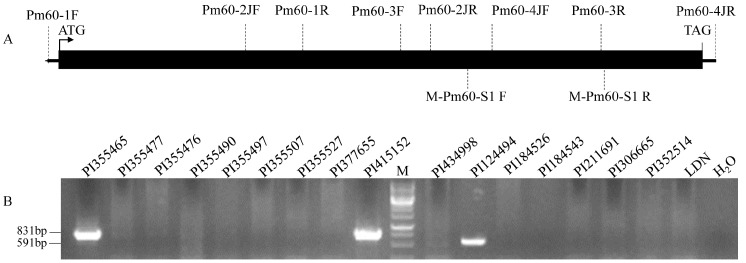
Marker-assisted screening of *Pm60* locus in wheat germplasm. (**A**), Locations of primers used for screening and sequencing of *Pm60* in this study. Black rectangle, the coding region of *Pm60*. (**B**), Agarose gel electrophoresis of PCR products amplified by marker *M-Pm60-S1*. LDN, tetraploid wheat cultivar Langdon (absence of *Pm60*); H_2_O, negative control; M, Thermo Scientific GeneRuler 1kb DNA Ladder.

*Pm60* alleles were previously isolated from *T. urartu* and *T. dicoccoides* accessions. To characterize whether *Pm60* alleles are present in other wheat species, we screened a collection of 238 wheat germplasm using the PCR-based molecular marker *M-Pm60-S1*, which covers the LRR regions and can differentiate the *Pm60*, *Pm60a*, and *Pm60b* alleles by the size of amplified products (Figure 1A). The accessions positive for the amplification of the marker *M-Pm60-S1* were selected to obtain the entire *Pm60* sequences (from start to stop codons) using four primer pairs amplifying overlapping fragments (Figure 1A and Table S2), which were developed based on the published nucleotide sequence around the *Pm60* locus in *T. urartu* [7]. The four PCR primer sets were also used for the sequencing of the *Pm60* alleles in the present study. The sequencing was performed by Sangon Biotechnology Company (Chengdu, China). Sequences were assembled and subjected to multiple alignments using DNAMAN software version 6.0 (Lynnon Biosoft, San Ramon, CA, USA). The final sequences were manually corrected, and SNPs were visually checked on a chromatogram to ensure their quality. 

### 2.4. Phylogenetic Analysis

The published *Pm60* alleles and homologous sequences from cereals were selected for phylogenetic analyses. The Pm60 homologs in the phylogeny tree were identified from the National Centre for Biotechnology Information (NCBI) database and the Ensembl Plants website. Multiple sequence alignments were performed with BioEdit [17], and a neighbor-joining tree was constructed using MEGA software by the bootstrap method with 1000 replicates.

### 2.5. Powdery Mildew Resistance Response

Five *Bgt* isolates including *Bgt#1-25, Bgt#15-9-4, Bgt#WJ, Bgt#SZS*, and *Bgt#GH* were used to test the response of wheat accessions harboring different *Pm60* haplotypes in the present study. Isolates *Bgt#1-25* and *Bgt#15-9-4* were kindly provided by Wenqi Shi from the Institute of Plant Protection and Soil Fertility, Hubei Academy of Agricultural Sciences. The isolates *Bgt#WJ* and *Bgt#SZS* were newly purified from bread wheat in the experimental fields of Sichuan Agricultural University and Sichuan Academy of Agricultural Sciences, respectively. The isolate *Bgt#GH* was purified from bread wheat in the greenhouse of Sichuan Agricultural University. Wheat accessions PI352335, PI355465, PI415152, and CItr7685 carrying HapI and accessions PI190949, PI113392, PI124494, and AS285 carrying HapII of *Pm60* were randomly selected for the powdery mildew resistance test. Wild emmer wheat accession G18-16 (*TdPm60*, highly resistant) [8] and Chinese Spring (highly susceptible) were used as controls. Wheat plants at the two-leaf stage were tested for resistance to powdery mildew as previously described by Li et al. [8]. In brief, the first leaf of a seedling plant was excised into three segments with approximately 2 cm in length and cultured in petri dishes harboring 8 g/L agar with 50 mg/L benzimidazole (Sigma-Aldrich, St. Louis, MO, USA). Dishes were inoculated with fresh spores of powdery mildew isolates and were transferred to a growth chamber with 75% humidity and kept in 20 °C/18 °C under a 16 h light/8 h dark photoperiod, respectively. Resistance results were recorded at 7 and 10 days post inoculation (dpi). Disease symptoms were recorded based on a 0–4 infection type (IT) scale as described by Wu et al. [14]. The IT scores were classified into two groups, of which 0–2 were considered as resistant (0 = immunity, 0 = necrotic flecks, 1 = highly resistant, and 2 = resistant) and 3–4 as susceptible reactions (3 = susceptible and 4 = highly susceptible).

## 3. Results

### 3.1. Distribution of Pm60 in Wheat Species

The *Pm60* functional marker *M-Pm60-S1* could amplify 591 bp for *Pm60a*, 831 bp for *Pm60*, or 1071 bp for *Pm60b*, whereas there was no amplification product for the absence of *Pm60* alleles [11]. As a result, 20 out of 238 accessions were positive for the *M-Pm60-S1* marker, whereas 218 showed no amplification (Table S1). The results showed that tetraploid wheat species, including *T. carthlicum, T. dicoccon, T. armeniacum, T. dicoccoides, T. durum*, and *T. turanicum*, were harboring the *Pm60* gene. Based on the size of the PCR products, we concluded that these tetraploid wheats may contain *Pm60* and *Pm60a* alleles (Figure 1B and Table S1). 

### 3.2. Sequence Variation of Pm60

The genomic sequence of the entire *Pm60* gene was amplified, using four overlapping primer pairs (Table S2), in the 20 tetraploid wheat accessions that may harbor *Pm60* alleles. The obtained sequences were compared with the published *Pm60* sequences and revealed three novel (HapI–III) haplotypes (Table S3, GenBank accession numbers OP893800-OP893802) and two known haplotypes MW375699 and MW375701 (Table S1), which were previously reported by Wu et al. [14]. HapI was detected in *T. dicoccon* (n = 6) and *T. turanicum* (n = 1) accessions; HapII was identified in *T. carthlicum* (n = 2), *T. durum* (n = 4), and *T. turanicum* (n = 4) accessions; HapIII was only found in *T. armeniacum* accession PI361859 (Table S1). 

**Figure 2:**
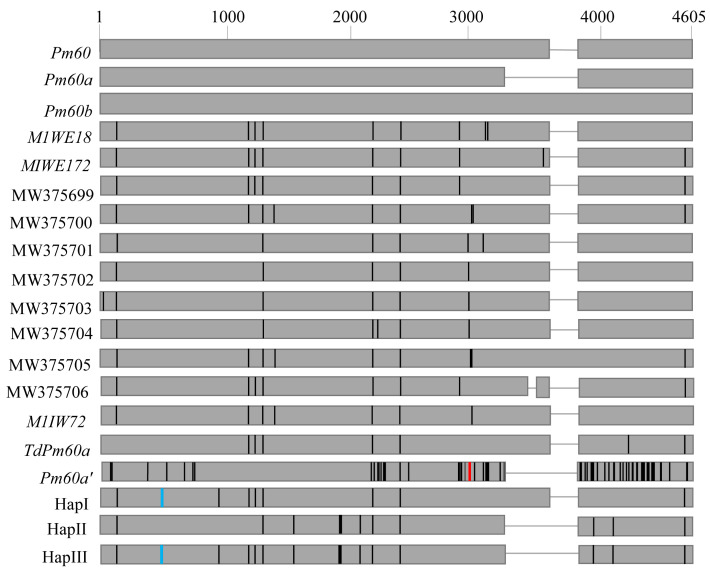
Schematic representation of sequence variation of *Pm60* in *Triticum* species. Black short vertical lines indicated SNP. Red transverse line indicated ‘TAG’ deletion in *Pm60a’*. Blue transverse lines showed ‘GCC’ InDel in the present study.

The size of the HapI sequence was 4,362 bp in length, which showed 99.75% similarity to *TuPm60* with eight SNPs and a GCC deletion. The sizes of the HapII and HapIII sequences were 4,215 and 4,122 bp, respectively, which both had a 240 nt deletion that was the same as the previously reported *TuPm60a*. The HapII and HapIII sequences were 99.71% and 99.56% identical to *TuPm60a*, respectively. Twelve and fifteen SNPs were identified in HapII and HapIII, respectively, as compared with *TuPm60a*. Only three SNPs and a GCC InDel were detected between HapII and HapIII (Table S3 and Figure 2). 

### 3.3. Phylogenetic Analysis of Pm60

**Figure 3:**
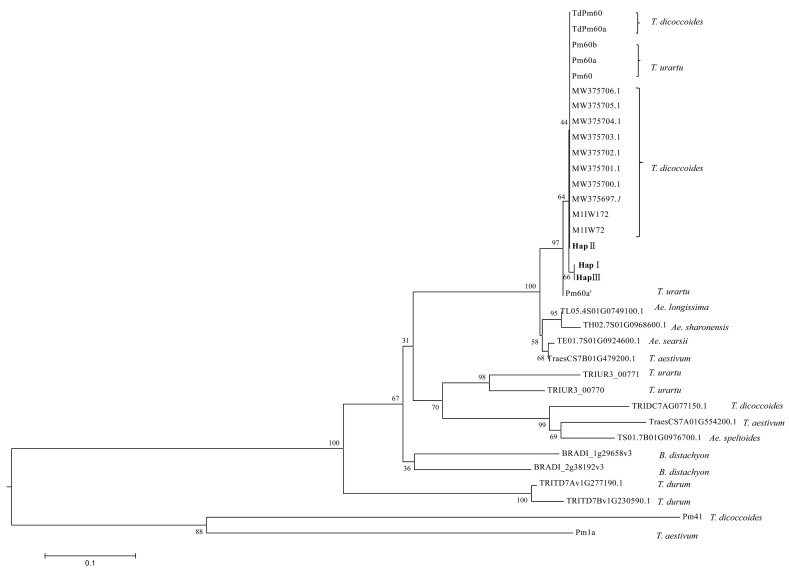
Phylogenetic analysis of Pm60 proteins and homologs from cereal species. Phylogenetic tree was constructed using the neighbor-joining method and implemented in the software MEGA7 with default settings. Branches are labeled with relevant plant species and protein accession number or Pm protein name. Numbers at nodes represent the percentage of replicated trees in which the associated taxa clustered together in the bootstrap test (1000 replicates). Wheat powdery mildew resistance proteins Pm1a and Pm41 were used as outgroup member. Three Pm60 haplotypes (HapI–HapIII) have been highlighted in bold.

To determine the phylogenetic relationship between Pm60 proteins and homologs in the annotated genome assemblies of domesticated wheat (*T. aestivum* and *T. durum*) and their wild relatives (*A. speltoides*, *A. longissima, A. searsii, T. urartu*, and *T. dicoccoides*) (Table S4), an unrooted tree was constructed using the neighbor-joining method (Figure 3). The results of the phylogenetic analysis indicated that all published Pm60 proteins from *T. urartu* accessions and wild emmer accessions as well as three Pm60 haplotypes in this study were classified into one clade. HapII was more closely related to the functional haplotype MlIW172. Four homologs (TraesCS7B01G479200.1, *Ae.searsii.*TE01.7S01G0924600.1, *Ae.longissima.*TL05.4S01G0749100.1, and *Ae.sharonensis.*TH02.7S01G0968600.1) were clustered very close to this reported Pm60. TRIUR3_00771 and TRIUR3_00770 from *T. urartu* acc. G1812, Ae.speltoides.TS01.7B01G0976700.1 from *Ae. speltoides*, TRIDC7AG077150.1 from *T. dicoccoides* acc. Zavitan, and TraesCS7D01G542200.1 from *T. aestivum* cv. Chinese Spring (CS) were classified on a cluster with longer branches than the Pm60 proteins. TRITD7Av1G277190.1 and TRITD7Bv1G230590.1 from *T. durum* acc. Svevo, and BRADI_1g29658v3 and BRADI_2g38192v3 from *Brachypodium distachyon*, were located as an outgroup in the phylogenetic tree.

### 3.4. Powdery Mildew Responses

To test if any of the newly identified *Pm60* haplotypes confers resistance to powdery mildew, a leaf infection test at the seedling stage was initially performed in eight randomly selected wheat accessions carrying either HapI or HapII of *Pm60* using different *Bgt* isolates. HapIII was not involved in the inoculation test due to the unavailable seeds of *T. armeniacum* accession PI 361859. All accessions were susceptible (IT = 3–4) to *Bgt* isolates *Bgt #1-25, Bgt#15-9-4, Bgt#WJ*, and *Bgt#SZS*, whereas PI190949, PI113392, PI124494, and AS285 carrying HapII of *Pm60* showed partial resistance to *Bgt#GH* (IT = 2; Table 1 and Figure S1). 

## 4. Discussion

The powdery mildew resistance gene *Pm60* isolated from the diploid *T. urartu* encodes a typical CC-NB-LRR protein that has at least three functional alleles, including *TuPm60*, *TuPm60a*, and *TuPm60b* [7]. *Pm60* were further identified in *T. dicoccoides*, which are the orthologs of *Pm60* from *T. urartu* [8,14]. In the present study, we have studied the diversity of *Pm60* genes in a large set of wheat accessions and found that *Pm60* alleles were not only present in wheat progenitor species *T. urartu* and *T. dicoccoides* but also distributed in cultivated tetraploid wheats. 

*Pm60* is an ancient gene present in *T. urartu* and *T. dicoccoides* species, where it shows a high level of presence/absence polymorphism [8,11,14]. *TuPm60* was the most prevalent one, and *TuPm60a*, *TuPm60a’*, and *TuPm60b* were the less prevalent alleles in *T. urartu* accessions [11]. The *T. urartu* accessions from Turkey were shown to have all forms of *Pm60* alleles. The wild emmer wheat population had functional *Pm60* alleles, which are only present in the natural populations of *T. dicoccoides* from the Southern Levant, and almost all of them belong to the *TdPm60* form [8,14]. The *TdPm60a* and *TdPm60b* alleles are rare and were identified only once [8,14]. In the current study, we found *Pm60* homologs present in 20 tetraploid wheats but not in the hexaploid wheat accessions tested. The presence of *Pm60* homologs in cultivated tetraploid wheats implicates that *Pm60* may be involved in wheat domestication events. The results of the sequence analysis revealed only three new *Pm60* haplotypes (HapI–HapIII). HapI was similar to *Pm60*. HapII and HapIII had a 240 nt deletion, which was the same as the previously reported *Pm60a*. Among them, HapI was present only in domesticated tetraploid wheats *T. dicoccom* and *T. turanicum*; HapII was found in tetraploid wheats *T. durum*, *T. turanicum*, and *T. carthlicum*; whereas HapIII was only identified in wild tetraploid *T. araraticum* accession PI361859. 

Phylogenetic analyses indicated that all proteins encoded by the *Pm60* gene were clustered into one clade, suggesting the evolutionary conservation of the *Pm60* locus in *Triticum* species. *T. dicoccun* (emmer wheat, A^u^A^u^BB) is the domesticated form of *T. dicoccoides* and is believed to have been domesticated probably in southeast Turkey. Cultivated emmer was later evolved into the free-threshing ears of durum wheat [18,19]. Therefore, we hypothesized that the HapI in emmer originated possibly from the northern populations of *T. dicoccoides* [20] with an uncharacterized *TdPm60* form. HapII in cultivated *T. durum* was likely derived from emmer wheat. HapIII in *T. araraticum* (A^u^A^u^GG) might be independently inherited from *T. urartu* by the polyploidization event. Further studies are still needed to test these hypotheses. 

A previous study showed that the nonfunctional allele *Pm60a’* with 58 SNPs and a 3-nucleotide deletion was detected in several susceptible *T. urartu* accessions, implying that these SNPs and deletion could be important for the resistance function [11]. In the present study, eight to fifteen SNPs were identified in between new *Pm60* haplotypes (HapI–HapIII) and functional *TuPm60a*. Besides, HapI and HapIII sequences had 3 nt deletion in the interdomain region between the CC and NB-ARC domains of *Pm60* as compared with HapII and *TuPm60*. HapII and HapIII sequences had a 240 nt deletion, which was the same as the previously reported *TuPm60a* [7]. We found that tetraploid wheat accessions carrying HapI or HapII sequences were susceptible to four tested *Bgt* isolates, whereas HapII (*Pm60a* allele) carrying lines showed partial resistance (IT=2) to *Bgt#GH*, suggesting that HapII may be functional. HapII differed from HapI by 10 SNPs and a 3 bp InDel as well as 240 nt deletion, which resulted in the loss of the two leucine-rich repeat motifs (Table S3). A recent finding demonstrated that deletion of the two leucine-rich repeat motifs in *TuPm60a* substantially narrowed the resistance spectrum [10]. It seems that these SNPs and a 3 bp InDel between HapII and HapIII are vital for the resistance function. However, we cannot rule out the possibility of the presence of other *Pm* genes in the HapII-carrying lines. 

Pyramiding of several resistance genes or resistance alleles has the potential to improve resistance in terms of durability and spectrum [21,22]. *TdPm60*, *Pm60*, and *Pm60b* have been successfully introduced from wheat donor species into common wheat using the durum wheat as a ‘bridge’ [3,8]. The developed introgression lines carrying different *Pm60* genes conferred high resistance to *Bgt* in hexaploid wheat genetic backgrounds, which facilitated the utilization of *Pm60* in wheat resistance breeding. A previous study observed a different resistance spectrum of *Pm60* alleles in *T. dicoccoides* (*MlIW172* and *MlWE18*) and in *T. urartu* (*Pm60, Pm60a*, and *Pm60b*) when inoculated with 20 *Bgt* isolates [14]. It seems that single *Pm60* cannot provide durable protection against wheat powdery mildew. Therefore, allele pyramiding of *Pm60* or pyramiding of *Pm60* with other disease resistance genes/alleles is valuable for breeding disease resistance wheat cultivars.

## Figures and Tables

**Table 1:** Reactions of wheat accessions with different *Pm60* haplotypes to five *Bgt* isolates at 10 days post inoculation.

□	□	HapI				HapII				□
*Bgt* Isolates	G18-16 (*TdPm60*)	PI352335	PI355465	PI415152	CItr7685	PI190949	PI113392	PI124494	AS285	Chinese Spring
*Bgt#1-25*	ND	4	4	4	3	4	4	4	3	4
*Bgt#15-9-4*	ND	4	4	4	4	3	4	4	3	4
*Bgt#SZS*	0	3	4	4	4	4	3	4	4	4
*Bgt#WJ*	0	3	4	4	4	3	4	4	4	4
*Bgt#GH*	0	4	4	3	4	2	2	2	2	4

Susceptible wheat cultivar Chinese Spring was used as the control. ND, not determined.

## Data Availability

Sequences were deposited in the National Center for Biotechnology Information GenBank under accession numbers OP893800-OP893802.

## Supplementary Materials

The following supporting information can be downloaded at: https://www.mdpi.com/article/10.3390/pathogens12020241/s1, Figure S1: Phenotypes of HapI- and HapII-carrying lines after inoculation with *Bgt#GH*. Table S1: A list of wheat germplasm tested for the presence of *Pm60* alleles; Table S2: Primers used in this study; Table S3: Haplotypes of the entire genomic DNA sequence of *Pm60* gene identified in the present study; Table S4: List of Pm60 homologs used in phylogenetic analysis.
