# Supplementary figures and images for: Haplotype Analysis Sheds Light on the Genetic Evolution of the Powdery Mildew Resistance Locus Pm60 in Triticum Species
Source: Pathogens. 2023 Feb 2;12(2):241. doi: 10.3390/pathogens12020241 (PMC9964976; doi:10.3390/pathogens12020241)

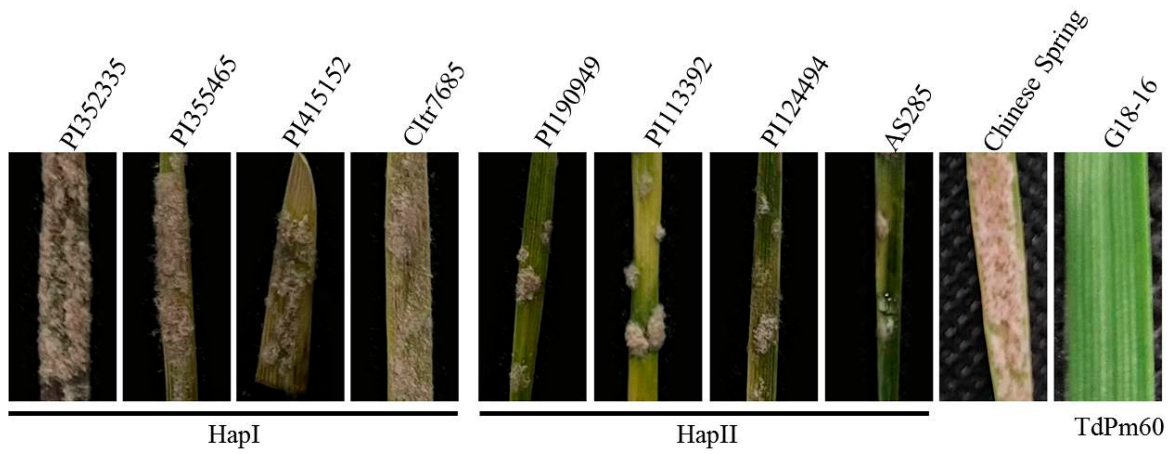

**Figure S1.** Phenotypes of HapI- and HapII-carrying lines after inoculation with *Bgt#GH*.

Supplement: Supplementary file 1 [file pathogens-12-00241-s001.zip › pathogens-2142991-supplementary.pdf]
